# Regulation of CD8 T cell by B-cells: A narrative review

**DOI:** 10.3389/fimmu.2023.1125605

**Published:** 2023-03-08

**Authors:** Tess Van Meerhaeghe, Antoine Néel, Sophie Brouard, Nicolas Degauque

**Affiliations:** ^1^ Department of Nephrology, Hôpital Erasme, Université libre de Bruxelles, Brussels, Belgium; ^2^ Nantes Université, INSERM, Center for Research in Transplantation and Translational Immunology (CR2TI), UMR 1064, Nantes, France; ^3^ Internal Medicine Department, Nantes University Hospital, Nantes, France

**Keywords:** CD8 lymphocytes +, B cell, crosstalk, regulation, auto-immune

## Abstract

Activation of CD4 T cells by B cells has been extensively studied, but B cell-regulated priming, proliferation, and survival of CD8 T cells remains controversial. B cells express high levels of MHC class I molecules and can potentially act as antigen-presenting cells (APCs) for CD8 T cells. Several *in vivo* studies in mice and humans demonstrate the role of B cells as modulators of CD8 T cell function in the context of viral infections, autoimmune diseases, cancer and allograft rejection. In addition, B-cell depletion therapies can lead to impaired CD8 T-cell responses. In this review, we attempt to answer 2 important questions: 1. the role of B cell antigen presentation and cytokine production in the regulation of CD8 T cell survival and cell fate determination, and 2. The role of B cells in the formation and maintenance of CD8 T cell memory.

## 1 Introduction

Cytotoxic CD8 T cells represent in the adaptive immune response to intracellular pathogens and various cancers. T cell responses can be divided into primary immune response and immunological memory. The primary immune response is the activation of naive CD8 T cells in response to a specific antigen presented by antigen-presenting cells (APCs) and their subsequent proliferation and differentiation into effector T cells. Immunological memory is created after the primary immune response and provides protection against subsequent attacks by the same pathogen. Memory CD8 T cells also need to be activated by APCs with costimulatory activity to regenerate effector CD8 T cells. Effector CD8 T cells express various cytokines such as interferon (IFN)-γ and tumor-necrosis factor (TNF)-α and the effector molecules, perforin, granzyme B (GZMB) and Fas-ligand (FasL). T-bet and Eomesodermin (EOMES) are essential transcription factors expressed in activated CD8 T cells that control their cytotoxic capacity (1, 2). During CD8 T cell response to infection, short-lived effector cells (SLEC) that will die by apoptosis during the contraction phase of the immune response express high level of T-Bet (3). On the other hand, EOMES is preferentially expressed in memory precursor effector cells (MPEC) and is required for the formation of memory CD8 T cells (4). B lymphocyte-induced maturation protein-1 (Blimp-1), a transcriptional repressor, controls SLEC formation and the transcription repressor B-cell lymphoma transcriptional repressor 6 (Bcl-6) is required for the generation of CD8 T-cell memory (5, 6). The balance between the different transcriptional factors determines the fate and efficient development of CD8 effector and memory T cells. At which level B-cells play a role in priming of CD8 T cells, differentiation into effector and memory CD8 T cells or recall of memory is still under investigation. However, several *in vitro* and *in vivo* models have attempted to unravel the mechanisms involved in the crosstalk between B cells and CD8 T cells.

## 2 Evidence of B-CD8 crosstalk from B-cell deficient and B-cell depletion models

### 2.1 Efficacy of B-cell depletion therapy in the treatment of autoimmunity beyond “simple” B cell depletion

Autoimmune diseases are usually classified as organ specific (e.g., type 1 diabetes (T1D), multiple sclerosis [MS], immune thrombocytopenia [ITP], bullous autoimmune dermatosis, primary membranous nephropathy) or multi-systemic (e.g., systemic lupus erythematosus, Sjögren syndrome, dermatomyositis, anti-neutrophil cytoplasmic antibodies (ANCA)-associated vasculitis).

**Figure 1:**
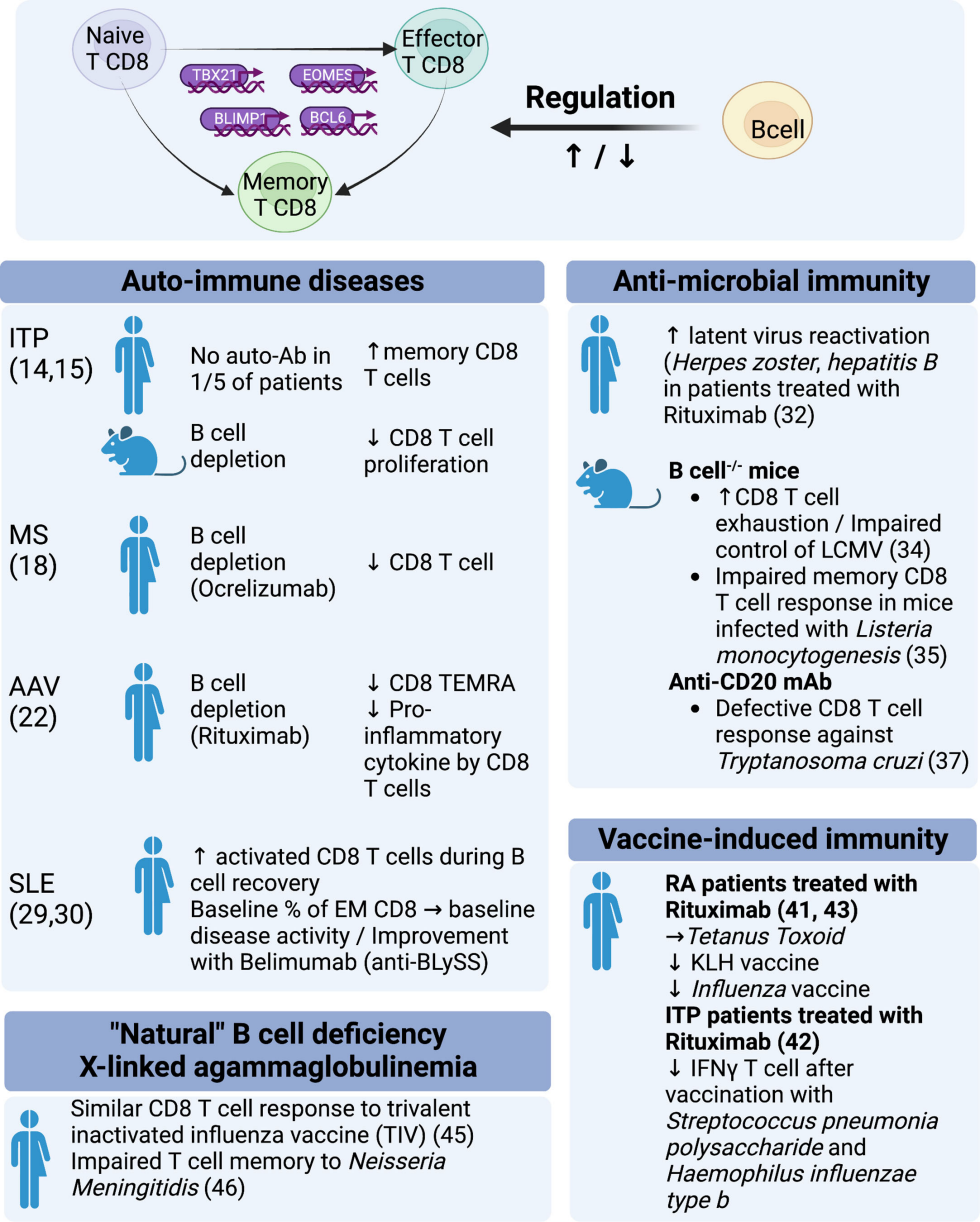
Regulation of CD8 function by B cells: evidence from experimental models and clinical observations.

The first step in understanding these diseases was the discovery of autoantibodies, which have proven to be very useful diagnostic tools and/or key effectors of damage *in vivo* and/or *in vitro*. However, it soon became apparent that even in the presence of disease-specific autoantibodies, T cells were actively involved in cellular and/or tissue damage. With the progress of knowledge, notably through the development of mouse models, it became possible to classify these entities as either “humoral,” i.e., autoantibody-mediated and therefore B-cell-dependent, or “cellular,” i.e., T-cell-mediated. Twenty-five years ago, the immunotherapeutic arsenal for these patients was still limited to immunomodulatory and/or pleiotropic immunosuppressive drugs (corticosteroids, disulone, antimalarials, interferon and cytotoxic agents such as cyclophosphamide, methotrexate or thiopurines, cyclosporine, an inhibitor of calcineurin), and to plasmapheresis, an antibody depletion therapy. Over the past 20 years, the development of truly targeted therapies, i.e., biologics, has transformed the management of several autoimmune diseases. B-cell targeted therapies have been shown to be biologically and/or clinically effective in diseases associated with autoantibodies but thought to be T cell mediated, such as rheumatoid arthritis (RA), MS, and, to a lesser extent, T1D. This apparent paradox has led to the realization that a dichotomous view of T cell or B-cell mediated autoimmunity is outdated. It is increasingly recognized that the role of B cells goes beyond the production of pathogenic autoantibodies, as already suggested by earlier experimental data obtained in MRL/lpr mice that develop nephritis despite the absence of serum autoantibodies (7). (**Figure 1**). The role played by B cells in maintaining a CD4 T autoimmune response has been well demonstrated in models of arthritis and diabetes (8). Several mouse models have shown that expression of CD80/CD86 costimulatory molecules (9), production of IL-6 (10) or IFN-γ (11) by B lymphocytes play important roles in experimental models of arthritis or encephalitis. More recently, data from mouse models and therapeutic B cell depletion in patients suggest that B cell may also promote a pathogenic CD8 T cell response in autoimmune diseases (**Figure 1**).

### 2.2 B-CD8 crosstalk, what can we learn from organ-specific autoimmune diseases?

ITP is characterized by autoimmune destruction of blood platelets. It is a prototypical antibody-mediated autoimmune disease. Response to B-cell depletion with Rituximab is frequent but inconsistent (~60%). One current explanation is that Rituximab refractoriness is characterized by the persistence of long-lived autoreactive plasma cells in the spleen and in other sites, the emergence of which may even be promoted by B-cell depletion itself (12, 13). However, alternative and nonexclusive mechanisms may be involved. One fifth of ITP patients have no detectable antibodies, which may suggest that some cases of ITP are not solely antibody mediated (**Figure 1**). Splenic expansion of effector memory CD8 T cells has been observed in Rituximab-refractory patients (14) (**Figure 1**). In a mouse model of ITP, B-cell depletion with Rituximab attenuated splenic CD8 T-cell proliferation and T-cell-mediated thrombocytopenia (15). These data illustrate the limitations of an overly dichotomous view of the pathogenesis of autoimmunity: B-cell depletion may have a paradoxical detrimental effect on pro-plasmacytic cells, whereas B-cell depletion may dampen a pathogenic CD8 T-cell response (16) (**Figure 1**).

Multiple sclerosis is another prototypical organ-specific T-cell-mediated autoimmune disease. As with other autoimmune diseases, most of the T-cell data have focused on CD4 T cells, particularly Th1, Th2, Th17, and Treg. Demonstration of the clinical benefit of B-cell depletion therapy has led to the consideration of B cells as a key architect of the immune response in MS (17). Rituximab has been shown to be effective in MS and paved the way for the development of Ocrelizumab, a comparable B cell depleting type 1 anti-CD20 antibody, which was approved by the FDA for MS in 2017. Recent long-term immunomonitoring data obtained in these patients demonstrate that B-cell depletion therapy impacts the CD8 T compartment (**Figure 1**). Abadessa et al. recently reported that Ocrelizumab decreased lymphocyte counts, with a predominant impact on CD8 T cells (18). Similarly, Capasso et al., analyzed immunoglobulin levels and CD4, CD8, and B-cell counts of patients receiving Ocrelizumab every six months for two years and observed continuous B-cell depletion with a progressive reduction in IgG and, notably, CD8 T cells. Importantly, the progression of disability was associated with a smaller reduction in CD8 cytotoxic T cells (**Figure 1**).

Finally, in T1D, CD8 T cells are considered key effectors of tissue damage. In non-obese diabetic (NOD) mice, B cells have been shown to promote the local CD8 T cell response, whereas B cell deficiency delays the development of diabetes (19) (**Figure 1**). In patients with newly diagnosed T1DM, B-cell depletion therapy with Rituximab was found to partially preserve beta-cell function during the first year (20).

### 2.3 B-CD8 crosstalk, what can we learn from systemic autoimmune diseases?

ANCA-associated vasculitis (AAV) and systemic lupus erythematosus (SLE) are multisystem immune-mediated diseases associated with pathogenic autoantibodies. In AAV, autoantibodies target neutrophil cytoplasmic proteins in a mutually exclusive manner: proteinase 3 or myeloperoxidase. In SLE, antibodies recognize numerous nuclear antigens such as dsDNA, histones, nucleosomes, ro/SSA52 (TRIM21), Sm protein and others. Humoral and cellular immune responses have been implicated in the tissue damage of AAV and SLE. In 2010, McKinney et al. performed transcriptional analysis of CD4 T cells, CD8 T cells, B cells, monocytes, and purified neutrophils (21). Somewhat unexpectedly, they identified a CD8 T-cell signature that predicted patient outcome in SLE and AAV, suggesting that CD8 T cells may play a key, but undervalued, pathogenic role in these diseases (**Figure 1**). In AAV, continuous B-cell depletion therapy with Rituximab (RTX) (500 mg IV every 6 months) was superior to azathioprine, a pleiotropic immunosuppressant (IS), as a remission maintenance therapy. How to reconcile the efficacy of B-cell depletion with transcriptomic data pointing to CD8 T cells is puzzling. During remission, we found that CD4 and Treg cell subsets were comparable in RTX- and IS-treated AAV patients, in contrast to CD8+ T cell subsets. RTX-treated AAV patients had a reduced frequency of CD8 CD45RA^+^CCR7^-^ T cells (Effector Memory expressing CD45RA; TEMRA) (**Figure 1**). Interestingly, cytokine/chemokine production of CD8 T cells was reduced in RTX-treated AAV patients but not in IS-treated AAV patients. In other words, B-cell depletion had a greater functional impact on CD8 T cells than an immunosuppressant that has a direct impact on T-cell biology (**Figure 1**). This paradoxical finding suggests that B cells play a role in the CD8 T cell response in AAV (22).

In SLE, several lines of evidence indicate that CD8 T cells are the effectors of tissue damage. In lupus nephritis, previous studies have reported that CD8 T cells are the predominant lymphocyte population in renal biopsy (23) and in urine (24). More recently, single-cell RNA sequencing of kidney samples from patients with lupus nephritis has identified several groups of CD8 T cells with high expression of granzyme and/or perforin (25) (**Figure 1**). Interestingly, such cytotoxic CD8 T cell signature was not prominent in the skin (26). One of the many immune disturbances seen in SLE patients is an expansion of CD4-CD8- α β T cells. Recent data strongly suggest that these double-negative T cells, which produce IL-17, are derived from autoreactive CD4-CD8- α β T cells (27, 28). A decade ago, two pivotal clinical trials using Rituximab (LUNAR, EXPLORER) failed to meet their primary endpoint, calling into question the relevance of targeting B cells in SLE. However, another B-cell-targeting therapy that was finally proven effective was Belimumab (monoclonal antibody directed against soluble B-cell stimulator [BLySS]), which was approved by the FDA for non-renal SLE in 2011 and for lupus nephritis in 2020. Data regarding the impact of B-cell targeted therapy on T cells are scarce in SLE. In 2007, Vallerskog et al. reported an increased frequency of activated CD8 T cells during B-cell recovery (29). More recently, Regola et al. reported that the initial proportion of effector memory CD8 T cells was associated with initial disease activity and improvement with Belimumab (30) (**Figure 1**). Recently, Obinutuzumab, a type II anti-CD20 that induces potent B-cell depletion, has shown promising efficacy in patients with lupus nephritis (31). Two phase III trials have been initiated (OBILUP, NCT04702256, and ALLEGORY, NCT04963296). Immunomonitoring data may shed light on the link between B cells and CD8 T cells in SLE, as was recently seen with Ocrelizumab in MS.

### 2.4 Antimicrobial immunity and vaccination

In clinical practice, it is now well documented that patients treated with anti-CD20 B-cell depleting therapy has a higher frequency of viral and opportunistic infections, suggesting T-cell immunodeficiency. In RA patients treated with RTX, the risk of herpes zoster is as high as in patients receiving a TNF-α or CTLA4-Ig inhibitor (Abatacept) (32). RTX-treated patients also have a 3-fold increased risk of developing *Pneumocystis Jirovecii* pneumonia compared with those receiving a TNF-α inhibitor (33). In addition, B-cell depletion therapy can lead to reactivation of hepatitis B associated with high morbidity and mortality. HBV screening and prophylaxis are of utmost importance in patients receiving RTX (34) (**Figure 1**).

In mice, several *in vivo* models have attempted to identify the pathways by which B-cell depletion affects not only humoral but also CD8 T cell mediated antimicrobial responses. Mice deficient in mature B cells infected with chronic strain *lymphocytic choriomeningitis virus* (LCMV) showed increased depletion of virus-specific CD8 T cell memory with impaired long-term viral control (35) (**Figure 1**). In line with these findings, a B-cell deficient (μMT^-/-^) mouse model infected with *Listeria monocytogenesis* showed impaired memory CD8 T cell responses. The authors demonstrated a normal phase of activation and expansion of Ag-specific CD8 and CD4 T cell responses in the absence of B cells, but there was a deeper contraction phase leading to a lower level of Ag-specific CD8 T cell memory. However, B cells played only a minimal role once memory was established (36) or in the case of infection with the acute strain of LCMV (37). Results in C57BL/6 mice treated with anti-CD20 also resulted in defective CD8 T-cell responses to the intracellular *Trypanosoma cruzi* parasite, characterized by a reduced level of functional, short-lived *Trypanosoma cruzi*-specific effector CD8 T cells. The expansion phase of the immune response was not affected, but the contraction phase was significantly shorter in the anti-CD20-treated mice. They demonstrated a lower frequency of interleukin-6 (IL-6) and interleukin-17A (IL-17A) producing cells when anti-CD20 was administered. Treatment of mice with recombinant IL-17A partially reversed the observed effect of B-cell depletion on CD8 T cells, indicating that IL-17A secretion by CD4 T cells may be important in the maintenance phase of the CD8 immune response (38). The role of IL-17A/IL-17RA was also demonstrated in a study by Xu et al., showing that IL-17A-deficient mice infected with *Listeria monocytogenes* had a deficient Ag-specific CD8 T cell response against the primary infection (39) (**Figure 1**). In humans, it has been suggested that B cell depletion may reduce the Th17 response in autoimmune diseases (17, 40, 41). How CD4 T cell influence the impact of B cell depletion on the CD8 T cell response remains to be delineated (**Figure 1**).

Patients treated with RTX can also show a reduced response to vaccination. The ability of patients treated with RTX to mount an efficient T cell response varies according to the nature of the vaccine. Response to *tetanus toxoid* (42) was similar in RA patients treated with RTX while a weaker response was observed to *pneumococcal polysaccharides* or to neoantigen keyhole limpet hemocyanin (KLH) vaccine (42). Impaired vaccine response was also noticed in patients treated with Rituximab for ITP: after vaccination with *Streptococcus pneumoniae polysaccharides* and *Haemophilus influenzae type b* there was a reduced antibody response and reduction of interferon-gamma-secreting T cells (43) (**Figure 1**). A reduced CD8 T cell vaccine response was also observed in RA patients treated with anti-CD20 upon *influenza* vaccination (44) and the authors postulated that direct type I interferon receptor signaling in B cells was important to support CD8 T cell expansion by upregulating MHC-I molecules (44). Reports on T cell responses after Sars-Cov-2 mRNA vaccination in patients treated with anti-CD20 showed strong S-specific CD8 T-cell responses and development of CD8 T cells with primarily an effector memory phenotype despite poor humoral responses (45). Obviously, B cell involvement in the induction of CD8 T cell response to vaccination may depend upon many parameters such as the nature of the antigenic target (protein vs. polysaccharides), the vaccine technology (Inactivated, Live-attenuated, subunits, recombinant, conjugate, viral vectors, toxoid, messenger RNA vaccines), adjuvants and route of administration.

X-linked agammaglobulinemia (XLA) is a primary immune deficiency resulting from the absence of Bruton’s tyrosine kinase (BTK), an intracellular signaling molecule essential for B cell differentiation and survival. XLA is characterized by recurrent bacterial infections due to the near-total absence of serum immunoglobulins and peripheral B cells. Liu et al. reported that CD8 cell responses to a trivalent inactivated influenza vaccine (TIV) were similar in XLA patients and healthy controls, suggesting that B cells are not involved in this CD8 vaccine response (46). However, Morales-Aza et al. have previously shown that despite a preserved memory response to influenza vaccination, XLA patients had an impaired naturally occurring T cell memory to *Neisseria Meningitidis*, a mucosa colonizing bacteria (47) (**Figure 1**). This result shows that the involvement of B cells in the T cell response is antigen and context dependent. Few data are available regarding CD8 T cells in XLA, which may shed light on our understanding of the role of B cells in the different types of CD8 T cell responses. It should be noted that BTK is also expressed in other immune cells (48).

## 3 Antitumoral immunity

CD8 T cells are the most important and powerful effectors in the anticancer immune response. Different therapeutic strategies based on enhancing CD8 T cell function are used in cancer treatment: immune checkpoint inhibitors (ICI) that enhance the function of cytotoxic T cells and adoptive T cell transfer with genetically engineered T cells. These treatment strategies have improved survival in patients with advanced cancer. However, the role of B cells in cancer immunity is increasingly recognized and there is evidence that tumor-infiltrating B lymphocytes (TIL-Bs) have an important impact in the elimination of malignant cells (49). This has been highlighted in studies showing the role of mature and differentiated B cells in response to ICI (50, 51). In addition, one study demonstrated that *ex vivo* expansion of CD8 T cells for adoptive T cell transfer was achieved using TLR-activated B cells. CD8 T cells acquired a unique IL-2Rα^high^ICOS^high^CD39^low^ phenotype and an altered metabolic profile, all dependent on the B cells present in the culture (52). Further evidence that B cells play a positive role in cancer control is derived from mouse models. In wild-type mice treated with an anti-CD20 monoclonal antibody before transfer of syngeneic B16 melanoma tumors, there was a significant expansion of tumor volume and two-fold increase in lung metastasis. Analysis revealed a reduction in effector memory (EM) and in IFN-γ–or TNF-α–secreting CD4 and CD8 T cells in B cell-depleted mice with tumors (53). In addition, mice treated with anti-IgM from birth are also more likely to develop virus-induced tumors due to the lack of effective T-cell immunity (54, 55). However, some evidence points to a negative regulation of B-cells in tumor immunity, as seen in mouse models where B-cell depletion results in a decreased risk of tumor growth and metastasis (56–59). This may be explained by the reduced IL-10 secretion by regulatory B cells, which enhances anti-tumor T cell responses (60).

Studies in humans demonstrate that the presence of a B-cell signature was associated with a better survival in sarcoma (61). In oropharyngeal squamous cell carcinoma, a high abundance of TIL-B and a higher density of B-cell and CD8 T-cell interaction predict an excellent prognosis. CXCL9 expression by TIL-Bs and their activated phenotype can potentially recruit more CD8 T cells and contribute to the maintenance of CD8 survival through secondary costimulation (62). In contrast, a study in human colon cancer showed delayed cancer progression and metastasis using RTX (57). B cells can play a paradoxical role in cancer: either they contribute to tumor cell destruction by enhancing T cell responses and *via* ADCC, or they contribute to tumor growth by promoting inflammation, angiogenesis or immunosuppressive mechanisms.

## 4 Mechanistic understanding of crosstalk between B cells – CD8 T cells

### 4.1 Cross-presentation by B cells to CD8 T cells

The mechanisms by which B-cells cross-present extracellular antigens to CD8 T cells have been investigated in several *in vitro* and *in vivo* models. B-cells possess antigen-presenting capacities and can present exogenous antigens *via* the class I pathway when taken up by receptor-mediated endocytosis (63). In NOD mice, a model of autoimmune diabetes, the singular loss of B – cells MHC class I subverted the conversion to clinical diabetes by controlling the expansion and development of self-reactive CD8 T cells (64). During the process of cross-presentation, Robsen et al. demonstrated that only *antigen-specific* B cells can stimulate CD8 T cells *in vitro* using oligomeric cognate antigen (OVA-hen egg, OVA-HEL) models. In the same study, they also demonstrated that the antigen can be effectively acquired *in vitro* or *in vivo* by immune stimulating complexes (ISCOMS) that sustained the primary expansion of CD8 T cells in OVA-HEL ISCOMS-immunized mice (65). These results may be important for vaccine therapeutic strategies to boost host immunity. However, the priming of CD8 T cells can only be achieved in the context of activated B cells. Indeed, previous studies have shown that immunization with naive resting B cells can induce T cell unresponsiveness in naive CD8 T cells (66). This could in part be explained by the low expression level of costimulatory molecules by naive resting B cells. The group of Mathieu et al. showed that CD40-activated B cells with CD40L with or without Toll Like Receptor (TLR) agonists (TLR9 agonist (CpG) or TLR4 agonist [Lipopolysacharide LPS]), can induce potent effector CD8 T cells in B6SJL mice. However, these B cells lack the capacity to induce CD8 memory T cells compared to dendritic cell (DC) immunization with a decreased expression of the transcription factors Bcl-6 in CD8 effector cells after CD40-B cell immunization compared to DC (67). In a mouse model of anti-tumor immunity, the TLR9 agonist CpG has been shown to enhance the CD8 T cell response *in vitro*, in a B cell and contact-dependent manner, resulting in an improved tumor growth control and a higher mouse survival (52). Tumor-specific murine CD8 T cells [derived from splenocytes of plem-1 T cell receptor (TCR) transgenic mice] were conditioned *in vitro* using TLR9 - activated B – cells. TLR-9-activated B cells played a central role in generating CD8 T cells with potent anti-tumor capacity *in vitro*, because depletion of B cells from the CpG-treated pmel-1 cultures led to deficient CD8 T cell responses in B57L/6 mice suffering from melanoma after injection. Therefore, CpG was used to booster T cells and B cells interaction during expansion *in vitro* and that the presence of activated B cells is crucial to induce efficient effector CD8 T cells (52).

**Figure 2:**
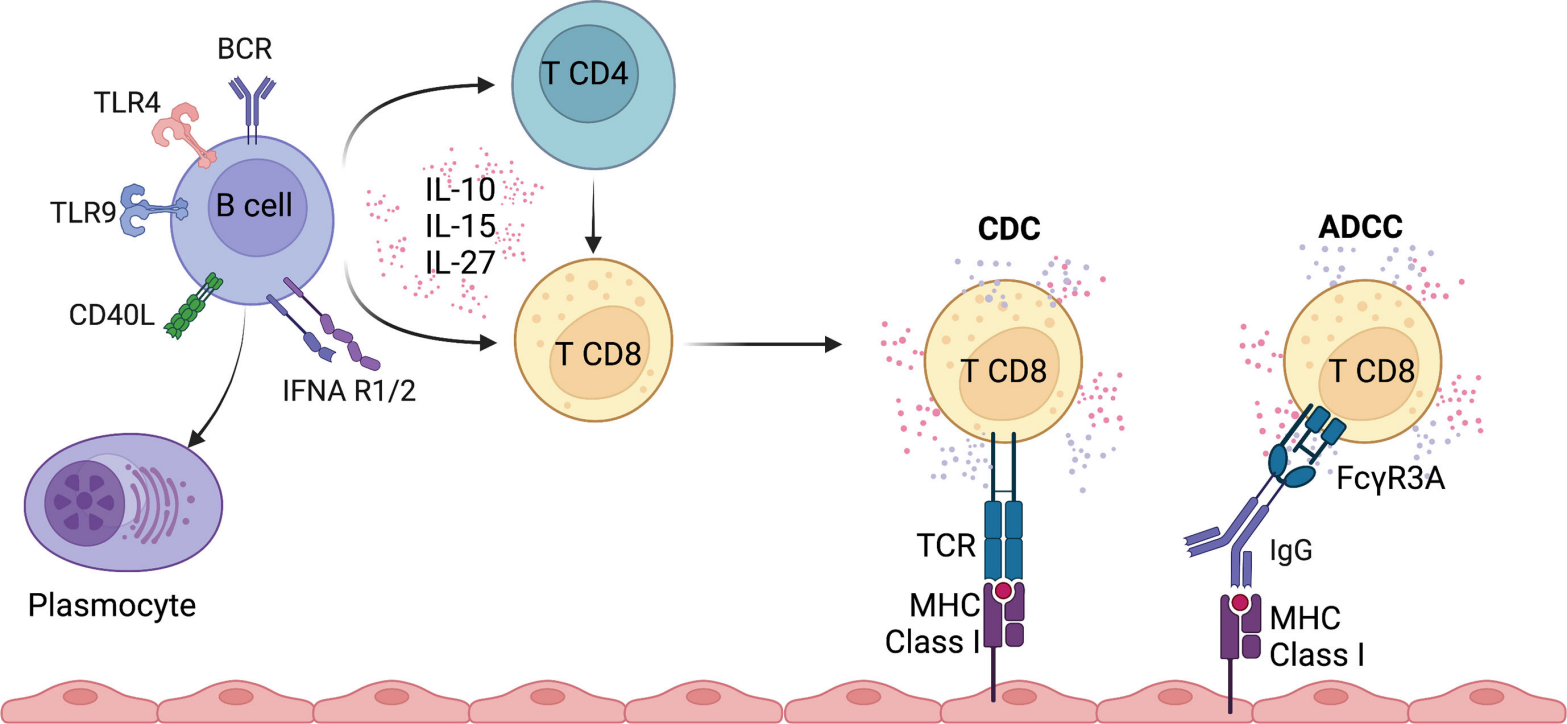
Overview of potential pathways involved in the regulation of CD8 T cell function by B cells.

The type of activation the B cell receives appears to condition the fate of the CD8 T cell response (68) (**Figure 2**), as hyporesponsive CD8 T cells are generated by LPS-activated B cells whereas efficient priming of CD8 T cells is achieved with anti-Ig plus anti-CD40 Ab-activated B cells. The fact B-cells need CD4 T-cell help to prime naive CD8 T cells has also been highlighted by Castiglioni et al. (69). The priming of CD8 T cells can occur either by the “three cell model” or the “four cell model.” Both models demonstrate that antigen-presenting B lymphocytes are conditioned by activated CD4 T cells either by cell contact or by soluble factors. Co-stimulation through CD40-OX40L seems to be of utmost importance and interestingly the effect of activated CD4 can be replaced by recombinant IL-4 (70). Using an HLA-A*0201-restricted, Flu M1:58-66-specific CTL model, investigators demonstrated *in vitro* that B cell can help in the survival and proliferation of antigen-specific CTL through the CD27/CD70 pathway. This could be a potential explanation how B cells regulate the expansion and contraction phase of the immune response. The crosstalk, however, in this model seemed independent of antigen presentation and induced the release of pro-inflammatory cytokines such as chemokines that target CD8 T cells expressing CXCR3 and/or CCR4 (71).

### 4.2 Engagement of FcγRIIIA expressed by CD8 T cell subsets, alternative activation by B cells

B cells could also indirectly activate CD8 T cells through Ig binding to FcγRIIIA (CD16), a receptor expressed by subsets of CD8 T cells and mediate antibody-dependent cellular cytotoxicity. Indeed, we have identified in kidney transplant recipients that effector memory expressing CD45RA CD8 T cells (TEMRA) express FcγRIIIA (72) (**Figure 2**). Upon FcγRIIIA engagement, a strong pro-inflammatory response is observed, with secretion of IFNγ and TNFα and release of cytotoxic molecules. The selective binding of CD8 TEMRA T cells to HLA class II molecules is observed in the presence of serum from immunized kidney transplant recipients with multiple HLA class II specificities and may therefore play a pivotal role in chronic humoral and cellular rejection. FcγRIIIA expression by CD8 T cells has also been reported in other chronic immune stimulation mainly in the context of chronic infection (hepatitis C (73), Epstein-Barr virus (74)). A recent study in severe Sars-Cov2 infection demonstrated the presence of highly cytotoxic CD16 T cells who provoke endothelial injury due to immune complex-mediated degranulation. The accumulation and activation of CD16 T cells were associated with a fatal outcome (75). These observations demonstrate that binding of Ig to its cognate antigen promotes an inflammatory immune response involving subsets of CD8 T cells.

### 4.3 Role of IL-27 production by B cells in the regulation of CD8 T cell function

IL-27 is a heterodimeric cytokine belonging to the IL-6/IL-12 family which binds to distinct IL-27R subunits (IL-27Rα and gp130) and induces downstream signaling through STAT1 and STAT3 pathways. IL-27 has a dual and opposite function with both pro- and anti-inflammatory properties. Upon combined stimulation of the TCR with IL-27, human naive CD8 T cells are rapidly activated into efficient and potent cytotoxic CD8 T cells (76). In contrast, IL-27 triggers IL-10 production in antigen-specific CD8 T cells during primary viral respiratory infection and thus promotes resolution of the inflammatory response (77). Under the same experimental conditions, memory CD8 T cells were shown to lose persistently their responsiveness to IL-27 associated with an inability to secrete IL-10. IL-27 is produced by B lymphocytes upon stimulation of the toll-like receptor (TLR), CD40 and IL-21 (78). Klarquist and colleagues demonstrated that IL-27 derived from B cells is an important cytokine for the primary but not the memory response of CD8 T cells after vaccination (79). Other evidence supporting the role of IL-27 produced by B cells is derived from anti-thymocyte globulin lymphoablation in mice, where B cells producing IL-27 induce reconstitution of the CD8 T cell compartment (80). The reconstitution of the CD8 T cell compartment was dependent of depletion-resistant memory CD4 T cells, CD40/CD154 interaction and the presence of intact B-lymphocytes (81). A recent study using a persistent clone of lymphocytic choriomeningitis virus (LCMV) revealed that B cells produce IL-27 and promote viral control *via* supporting the accumulation of CD8 and CD4 T cells (68).

### 4.4 Role of IL-15 production by B cells in the regulation of CD8 T cell function

IL-15, a member of the IL-2 family, is an important homeostatic and inflammatory cytokine contributing to the activation, proliferation and maintenance of memory CD8 T cells (70). In a broad variety of inflammatory environments, dendritic cells, monocytes and macrophages are important sources of IL-15. IL-15 is sufficient for TCR-independent activation and migration of pre-existing memory CD8 T cells in patients with acute hepatitis A (82, 83). Stimulation with IL-15 promotes the acquisition of cytotoxic functions and, to a lower extent, the production of pro-inflammatory cytokines IFNγ by memory CD8 T cells (84, 85) (**Figure 2**). Interestingly, B cells from patients with MS produce higher levels of IL-15 compared to controls and enhance the cytotoxicity of CD8 T cells by increasing granzyme B production (86). Moreover, the CD8 T cells were more responsive to IL-15 and had a higher migratory capacity across the blood-brain barrier. IL-15 secretion by B cells was induced upon CD40L-mediated activation, but stimulation through TLR ligands or B cell receptor (BCR) activation did not lead to increased secretion (86). Finally, B cell-activating factor (BAFF) suppressed IL-15 production by B cells in mouse models of autoimmune diseases (lupus-like and experimental autoimmune encephalomyelitis) (87), suggesting that BAFF inhibition could be associated with IL-15 inhibition to prevent accumulation and activation of CD8 T cells. Overall, IL-15 secretion by B cells could potentiate the activation of peripheral and *in situ* memory CD8 T cells.

### 4.5 Control of CD8 function by regulatory B Cells

A subset of B cells, the so-called regulatory B cells (Breg), has been shown to suppress immune response and support immune tolerance. More than two decades ago, Bennett et al. demonstrated that B cells can directly tolerize CD8 T cells after syngeneic injection of OVA_257-264_ coated B cells (88). To date, the Bregs are primarily characterized by their regulatory mechanisms involving secretion of IL-10, IL-35, expression of transforming growth factor (TGF)-β, programmed cell death ligand 1 (PD-L1) and production of GZMB (89). The literature highlights that regardless of their developmental stage, B cells can acquire a suppressive function upon appropriate stimulation and putative markers of Breg have been identified, such as CD9, CD5, TIM1 (90–93). The suppressive activity of Breg subsets is tested almost exclusively by *in vitro* analysis of CD4 T cell proliferation but their regulatory function toward CD8 T cells has been very poorly explored. As mentioned previously, LPS-activated B cells failed to trigger proliferation, secretion of pro-inflammatory cytokines and cytotoxic response of CD8 T cells, and the induction of hyporesponsive CD8 could be partially reversed with blocking anti-TGF-β1 antibody (94). Natural protection from type 1 diabetes in nonobese diabetic (NOD) mice was associated with an increased number of IL-10 producing B cells. IL-10 producing Bregs induce a tolerogenic dendritic cell state that suppresses pathogenic CD8 T cells. The stimulation of TLR4 pathway can restore IL-10 production by B cells in type 1 diabetic mice, leading to diminished CD8 T cell responses (95). Recently, the release of the neurotransmitter gamma-aminobutyric acid (GABA) by B cells was shown to limit the activation of CD8 T cells and macrophages and thereby impaired anti-tumor T cell responses (96). Stimulation with toll-like receptor or BCR induces the secretion of GABA by B cells, and the treatment with GABA of CD8 T cells limits their proliferation and the secretion of IFNγ and TNFα pro-inflammatory cytokines. The field of regulatory B cells is still in its infancy and further work is needed to define their ability to limit the primary or memory/recall CD8 response and to assess their impact on CD8 subset differentiation (naive vs. effector memory (EM) vs. TEMRA vs. tissue-resident memory T cells (TRM)).

## 5 Conclusion

Evidence is accumulating to support the role of a cross-talk between B and CD8 T cells in controlling infectious disease, tumoral pathologies and the development of autoimmune disorders (**Figures 1**, **2**). Studies with B cell depleting agents demonstrate a link between B lymphocytes and CD8 T cell responses *in vivo*. B cells are thought to be key players in the amplification of self-reactive CD8 T cells because B depletion induces proliferation and memory development defects in CD8 T cells. However, B cells do not seem to play a role in the initial activation of cytotoxic lymphocytes as shown in different infectious disorders or autoimmune diabetes (36, 38, 64). How B and CD8 T cells interact still needs further investigation but cross presentation of antigen *via* MHC class I and of secretion of different cytokines may play a central role. Cross presentation of exogenous antigens, under resting conditions induces CTL tolerance, but under inflammatory conditions can lead to CTL activation *via* the CD40 pathway. Within the cytokines of importance IL-2, IL-4, IL-15, IL-27, IL-17 and the IFN type I pathway seem to orchestrate the B-T cell interaction. Some evidence also points to the central role of CD4 T cells supporting B and CD8 T cell interaction.

The identification of the different pathways responsible for an effective B-CD8 T cell interaction is of utmost importance to understand disease pathology and is crucial for the development of effective vaccine and antitumoral strategies. The way is paved to unravel the complex interplay between B and CD8 T cells and between B cells and the different subtypes of CD8 T cells.

## Author contributions

TV, AN, SB and ND wrote the review, contributed to manuscript revision, read, and approved the submitted version.
